# Wafer-scale bioactive substrate patterning by chemical lift-off lithography

**DOI:** 10.3762/bjnano.9.31

**Published:** 2018-01-26

**Authors:** Chong-You Chen, Chang-Ming Wang, Hsiang-Hua Li, Hong-Hseng Chan, Wei-Ssu Liao

**Affiliations:** 1 Department of Chemistry, National Taiwan University, Taipei 10617, Taiwan

**Keywords:** bioactive substrate, chemical lift-off lithography, patterning, self-assembled monolayer

## Abstract

The creation of bioactive substrates requires an appropriate interface molecular environment control and adequate biological species recognition with minimum nonspecific attachment. Herein, a straightforward approach utilizing chemical lift-off lithography to create a diluted self-assembled monolayer matrix for anchoring diverse biological probes is introduced. The strategy encompasses convenient operation, well-tunable pattern feature and size, large-area fabrication, high resolution and fidelity control, and the ability to functionalize versatile bioarrays. With the interface-contact-induced reaction, a preformed alkanethiol self-assembled monolayer on a Au surface is ruptured and a unique defect-rich diluted matrix is created. This post lift-off region is found to be suitable for insertion of a variety of biological probes, which allows for the creation of different types of bioactive substrates. Depending on the modifications to the experimental conditions, the processes of direct probe insertion, molecular structure change-required recognition, and bulky biological species binding are all accomplished with minimum nonspecific adhesion. Furthermore, multiplexed arrays via the integration of microfluidics are also achieved, which enables diverse applications of as-prepared substrates. By embracing the properties of well-tunable pattern feature dimension and geometry, great local molecular environment control, and wafer-scale fabrication characteristics, this chemical lift-off process has advanced conventional bioactive substrate fabrication into a more convenient route.

## Introduction

Patterning on the micro- to nanoscale plays a key role in modern scientific and engineering research fields. Particularly, the creation of bioactive surfaces with well-defined geometries has drawn lots of attention due to its versatile applications in chemistry, biology, biophysics, and bioengineering [1–4]. In order to achieve the feature size and procedure convenience requirements that satisfy the specific needs for different applications, the adaptation of appropriate micro/nanofabrication approaches is necessary. The fact that optical lithography allows for large-area fabrication provides an accessible route to bioactive substrate manufacturing but is limited by light diffraction when very fine control is required. On the other hand, scanning beam-based lithographic techniques allow for highly controllable fine feature dimensions but are restricted by a much slower production speed for large-area patterns. Combinational and complimentary approaches are therefore widely adopted in the fabrication of bioactive substrates, which also provide the advantages of convenient process, economical operation, large-scale reproduction, and high resolution control.

Conventional strategies to fabricate bioactive substrates rely on the creation of different local molecular environments that are suitable for subsequent biological species attachment. For example, the supported lipid bilayer system has been widely used for many biophysical studies due to its mimetic property toward cell membranes [5–6]. These platforms often use a flat glass surface to support the formation of lipid bilayers via vesicle fusion. With the combination of microfluidic devices and lithographic techniques, the creation of multiplexed arrays has been achieved and delivers applications such as protein–ligand binding study and biological species sensing [7–9]. The other widely adopted system, the self-assembled monolayer (SAM), provides ease of operation and high stability under ambient conditions [10–11]. These approaches utilize the self-arrangement of silane or thiol molecules on silica or Au surfaces, which has been proven as a convenient route for the fabrication of functional surfaces toward versatile targets [12–16]. In addition to direct surface modification, the versatility of anchoring molecule tail groups provides further functionality choices which benefit the fabrication of variable recognition arrays [17–20]. It should be noted that the establishment of a proper surface environment for biological species recognition requires appropriate molecular moiety selection not only for better ligand–target interaction, but also lower non-specific binding of objects. A highly controllable molecular manipulation over large area surfaces is consequently highly sought after in bioactive substrate fabrication.

The recently developed chemical lift-off lithography (CLL) technique is a straightforward approach to create distinct regions carrying dissimilar surface properties for a variety of applications [21]. With conformal contact-induced reactions, the top layer Au–Au bond breakage on an alkanethiol SAM covered Au substrate leads to the exposure of fresh Au toward the exterior environment. These vacancies can therefore be fulfilled with different molecules, e.g., ligands, and the substrate is active for biological responses. Comparing to conventional lithographic stamping processes which use mobile inks, this approach solves the problems of molecular lateral diffusion and gas transport obstacles. The fabricated feature resolution can therefore be accomplished down to the sub-30 nm scale with high pattern fidelity [22–24]. Furthermore, the great stability of the created pattern, which could be attributed to the atomic scale step-edge from the lift-off process, allows for long-term usage of the generated platform [25–26]. It is important to note that the lift-off process creates a local molecular environment that is very unique and highly suitable for the insertion of bioactive probes [26–27]. Due to the incompleteness of the contact-induced reaction, the post lift-off region represents an unusual behavior compared to other SAM systems. Here, the lift-off process creates a diluted molecular matrix environment which is expected to be due to partial Au thiolate removal in SAMs. With the appropriate adjustment of the experimental conditions, this “diluted artificial defect-rich” matrix provides an abundance of opportunities to create different bioactive substrates via a straightforward one-step SAM defect control. Compared to conventional biological platform generation, this matrix provides the advantages of wide probe compatibility, minimized nonspecific biospecies adhesion, versatile platform creation, precisely controlled biomolecule positioning, and straightforward operation. To investigate the capability of the lift-off process to fabricate various bioactive substrates, probes with different molecular behavior and multiplexed array construction are tested. The unique surface environments created are also applied to execute patterns with diverse geometries, a wide range of feature dimensions, and structure repeatability, which are important in practical bioactive substrate assembly. To further expand this approach toward biologically important probe-anchored multiplexed patterns, a novel integration of microfluidics with CLL operation is also introduced. In addition, this strategy enables straightforward creation of wafer-scale bioactive substrates via one-step surface defect-rich matrix generation, which greatly advances the creation of a biofunctional platform manufacturing for practical uses.

## Experimental

**Materials.** 11-Mercaptoundecanol (MCU), 1-undecanethiol (UT), tri(ethylene glycol) undecanethiol (TEG), hexaammineruthenium(III) {[Ru(NH_3_)_6_]^3+^} chloride and 4-(2-hydroxyethyl)piperazine-1-ethanesulfonic acid (HEPES) were purchased from Sigma-Aldrich (St. Louis, MO, USA). Biotin-terminated hexa(ethylene glycol)undecanethiol was purchased from Nanoscience Instruments Inc. (Phoenix, AZ, USA). Streptavidin was purchased from Invitrogen Inc. (Carlsbad, CA, USA). FITC-labelled antistreptavidin antibody was purchased from Abcam Inc. (Cambridge, MA, USA). Tris(hydroxymethyl)aminomethane (TRIS) and tris(2-carboxyethyl)phosphine (TCEP) were obtained from Acros Organics (Geel, Belgium). Bovine serum albumin (BSA), and 10× phosphate buffered saline (PBS) containing 1.37 M NaCl, 0.027 M KCl, 0.10 M Na_2_HPO_4_, and 0.018 M KH_2_PO_4_ were purchased from Bioman Scientific Co., Ltd (Taipei, Taiwan). Deionized water (>18 MΩ·cm) was obtained from the ELGA PURELAB classic system (Taipei, Taiwan).

**Oligonucleotide sequences.** Oligonucleotides purified by HPLC were purchased from PURIGO Biotechnology Co., Ltd. (Taipei, Taiwan). The oligonucleotide sequences are listed in the following:

1. Hg^2+^-specific probe (30 bp): 5’ HS-(CH_2_)_6_-ACT CAT GAT TCT TTC TTC CCC TTG TTT GTT-FAM-3’ (FAM: carboxyfluorescein)
2. adenosine-specific probe (35 bp): 5’ HS-(CH_2_)_6_-ACT CAT GAA CCT GGG GGA GTA TTG CGG AGG AAG GT-FAM-3’
3. cocaine-specific probe (46 bp): 5’ HS-(CH_2_)_6_-ACT CAT GAG GGA GAC AAG GAA AAT CCT TCA ATG AAG TGG GTC TCC C-FAM-3’
4. target DNA-specific probe (42 bp): 5’ HS-(CH_2_)_6_-GCG ACT GGG ATT AAA TAA AAT AGT AAG AAT GTA TAG CCC AGT-FAM-3’
5. target DNA (33 bp): 5’-GCT ATA CAT TCT TAC TAT TTT ATT TAA TCC CAG-3’

Before tethering the thiolated probes onto CLL-treated substrates, 5 μL of the 10 μM probe solution was first mixed with 5 μL of 20 μM reducing agent (TCEP) in 25 mM TRIS buffer (150 mM NaCl, pH 8.2) for 30 min. The probe solution was then diluted to a final concentration of 0.5 μM in 25 mM TRIS buffer (150 mM NaCl, pH 8.2), and kept in the dark before use.

**Bioactive substrate preparation by the CLL process.** Silicon substrates (Mustec Corp., Hsinchu, Taiwan) with 100 nm thick Au and 5 nm chromium adhesive layers were prepared by thermal evaporation. The Au substrates were immersed in 0.5 mM MCU or TEG ethanolic solution for >6 h to form self-assembled monolayers. After SAM formation, the substrates were washed with ethanol to remove excess thiol molecules, and blown dry with nitrogen gas. Polydimethylsiloxane (PDMS) stamps with various patterns were fabricated by standard photolithography-created masters. A 10:1 mass ratio of SYLGARD 184 silicone elastomer base and curing agent (Dow Corning, Midland, MI, USA) was thoroughly mixed, degassed under vacuum, cast onto master molds, and cured on an aluminum-top hot plate at 100 °C overnight. The PDMS stamps were separated from the master molds, sequentially rinsed with acetone and isopropanol, and then blown dry with nitrogen gas. The prepared stamps were activated by 40 s of oxygen plasma exposure at a power of 18 W with 0.5 mbar oxygen flow. Thereafter, the stamps were conformally sealed onto the SAM-modified substrates to initiate a contact-induced reaction for typically 60 min. After separating the contact-sealed stamps from the Au substrates, 0.5 μM thiolate probe solutions were quickly dropped onto the surfaces to anchor the biological probes into the post-chemical lift-off regions. After typically 1 h of incubation, the substrates were gently rinsed by deionized water, immersed in buffer solution, and stored at 4 °C in the dark.

**Fluorescence image recording.** An epifluorescence microscope (Axio Imager, M2, Carl Zeiss Microscopy, Jena, Germany) equipped with an X-Cite^®^ 120 LED (Lumen Dynamics Group Inc., Mississauga, Canada) lamp and a fluorescence filter set with excitation and emission wavelengths of 480 ± 15 nm and 535 ± 20 nm, respectively, was used. The relative fluorescence intensity was processed with the rectangle function in ZEN 2012 (blue edition) Service Pack 2 software (Carl Zeiss Microscopy, Jena, Germany).

**Atomic force microscopy characterization.** The bioactive substrate fabrication process was step-wisely characterized by the tapping mode atomic force microscopy (AFM, Dimension Fastscan, Bruker Nano Surfaces, Hsinchu, Taiwan). Topographic AFM images were collected using a silicon cantilever with a spring constant of 48 N/m and a resonance frequency of 190 kHz (Nanosensors, Neuchatel, Switzerland). The substrates were gently rinsed by deionized water and carefully blown dry with nitrogen gas before characterization.

**Cyclic voltammetry (CV) measurements.** Electrochemical experiments were performed on the CH Instruments 627A electrochemical analyzer in a three-electrode system consisting of the prepared substrate (with an exposed area of 0.28 cm^2^), an Ag/AgCl (3 M KCl) reference electrode, and a Pt wire counter electrode. CV measurements were carried out with 1 mM [Ru(NH_3_)_6_]^3+^ in 25 mM TRIS buffer (pH 7.4) at a scan rate of 100 mV/s.

**Target capture.** For binding partner recognition, the substrates were rinsed with 25 mM TRIS buffer (150 mM NaCl, pH 7.4) in advance. Target DNA solutions in 25 mM TRIS buffer (150 mM NaCl, pH 7.4) was thereafter dropped onto the target DNA-specific probe-modified substrates for a 5 min incubation period. For sandwich-like array signal reporting, biotinylated thiol-patterned substrates were first exposed to 10 mg/mL BSA for 5 min to reduce nonspecific protein adsorption. The patterned surfaces were then treated with 50 μg/mL streptavidin solution for 20 min followed by 20 min of 10 μg/mL FITC-labelled antistreptavidin antibody incubation. These substrates were all rinsed with deionized water between single steps.

**Multiplexed biological probe-anchored platform.** After 40 s of oxygen plasma treatment, a flat PDMS stamp was conformally sealed onto a MCU SAM modified Au substrate for 60 min. After the stamp removal, the Au substrate was conformally sealed to another plasma-treated stamp rendering 20 μm microchannels. Three different thiolated probe solutions (5 μM in 25 mM TRIS buffer, 150 mM NaCl, 1 μM TCEP, pH 8.2) were introduced into separated channels for 60 min biomolecule insertion. The microchannel stamp was then quickly separated from the Au substrate in a deionized water bath. Finally, the Au surface was gently rinsed with deionized water and 25 mM TRIS buffer (pH 7.4) before further use.

## Results and Discussion

**Figure 1:**
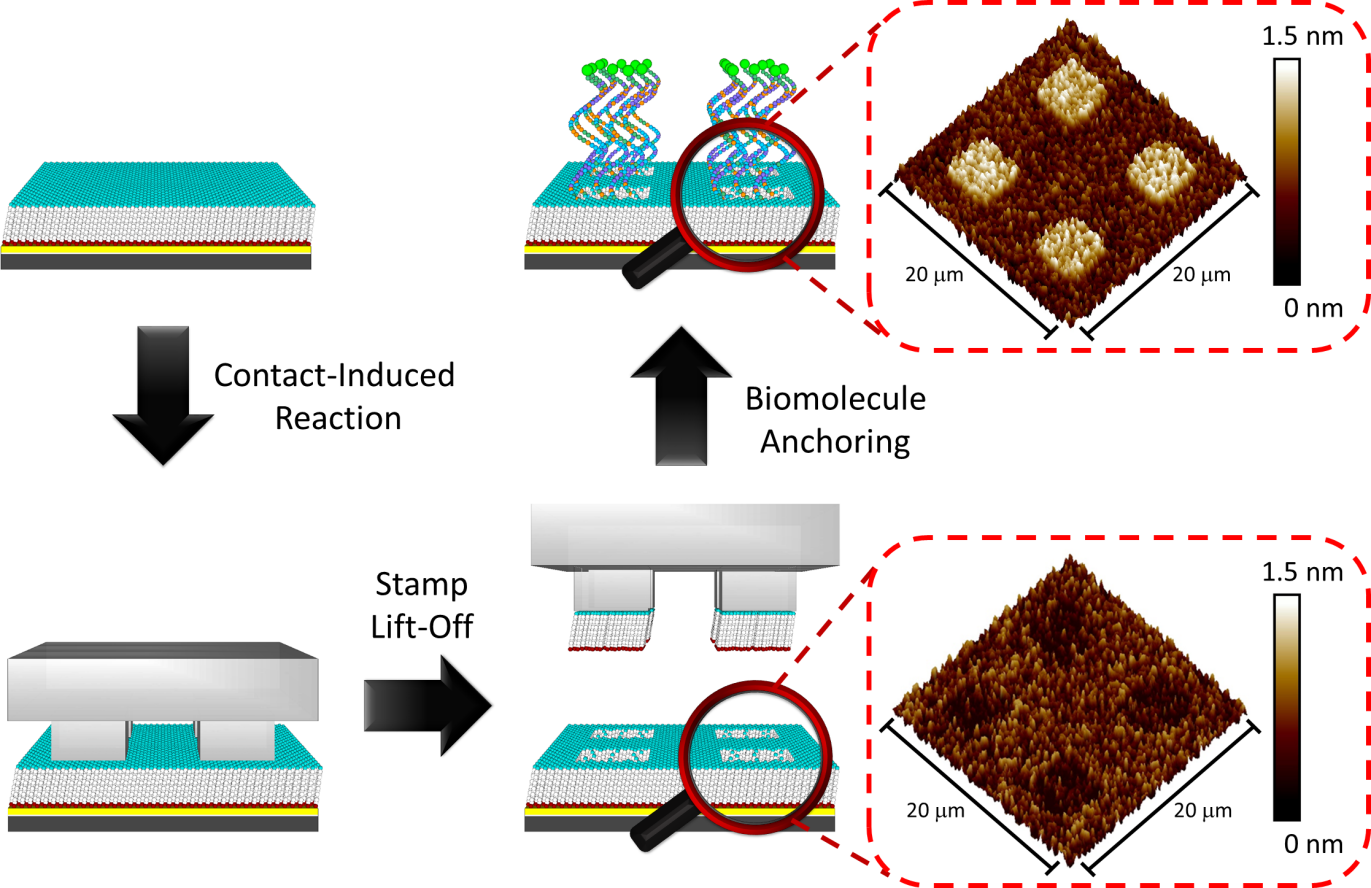
Schematic illustration and corresponding topographic AFM images of biological-probe-patterned surface fabrication using the chemical lift-off lithography (CLL) process.

The use of CLL for the fabrication of bioactive substrates depends on several governing factors to create a proper surface environment for biomolecule recognition. Hydrophilic group-terminated alkanethiol molecules provide a strong interaction toward an oxygen-plasma-treated PDMS surface, which is the best option for generating SAM ruptures by substrate top layer Au–Au bond breakage [11,28–32]. Another important task in the CLL operation is the choice of an appropriate molecular matrix, where the influence of molecular-level steric effects dominates the insertion of biological probes or the corresponding partner recognition [27]. An eleven carbon-chain-based 11-mercaptoundecanol (MCU) molecule is therefore selected in this study due to its great self-assembly behavior on Au and the suitable hydroxy-tail group toward the activated PDMS surface for the contact reaction [18–19,24,27]. A schematic illustration of the standard CLL operation is demonstrated in Figure 1, including PDMS activation, contact-induced reaction, lift-off steps, and biomolecule anchoring. It should be noted that the conformal contact reaction requires no external pressure, and the lift-off operation is performed under ambient conditions. As shown in the AFM images of Figure 1, the SAM-modified Au surface reveals a depressed square pattern after CLL operation when a PDMS stamp with a protruding square pattern is used. This depression region represents a freshly exposed Au area, which provides a position for subsequent biological probe insertion. For visualization, thiolated molecules of longer molecular length are used and give an inversed protruding topographic image, depicting successful probe insertion.

**Figure 2:**
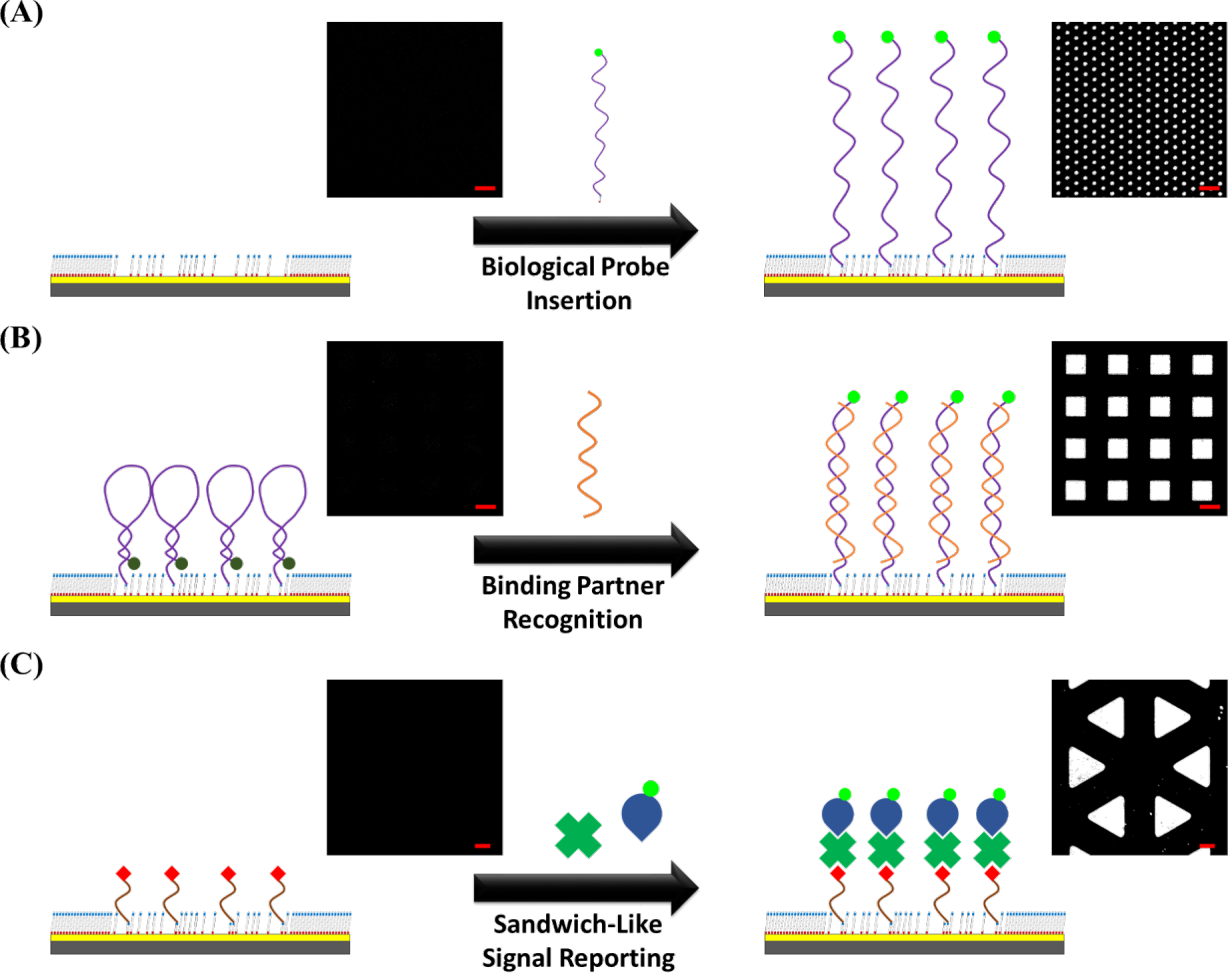
Different types of bioactive surfaces fabricated by the chemical lift-off lithograpy (CLL) process with corresponding fluorescence images. (A) Direct signal reporter-labelled probe insertion: the CLL-treated substrate before (left) and after (right) FAM-labelled Hg^2+^-specific probe insertion. (B) Binding partner recognition triggered probe structure change inducing signal readout: the CLL-treated substrate anchoring with the FAM-labelled hairpin-structured DNA probe before (left) and after (right) target DNA introduction. (C) Sandwich-like signal reporting with the inserted probe: the CLL-treated substrate anchoring with the biotinylated thiol before (left) and after (right) the conjugation with streptavidin and FITC-labelled antistreptavidin antibody. The scale bars are 20 μm.

The biological species filled inside the freshly created Au regions in this approach should maintain their activity toward targets for perspective practical applications. For investigation convenience, a fluorescence signal read-out based approach is applied and depicted via microscopic images. Three different categories of biological probes and their binding partner recognition capability are studied, including direct signal reporter-labelled probe insertion, binding partner recognition triggered probe structure change, and sandwich-like signal reporting with inserted probes, as shown in Figure 2. A thiolated six carbon chain based nucleotide probe with a carboxyfluorescein (FAM) moiety labelled tail is selected in the most straightforward probe direct insertion approach for demonstration (Figure 2A). The anchoring of this probe on the surface presents a high fluorescence image contrast, which indicates sufficient space and proper molecule orientation on the platform. This observation is attributed to the incomplete contact-induced reaction resulting in alkanethiol molecule residuals in the post lift-off region, allowing for inserted molecule self-orienting and reduced nonspecific probe–substrate adhesion. In Figure 2B, the second type of bioactive substrate fabrication approach relies on a labelled probe attachment with subsequent binding partner recognition induced signal output. A FAM-labelled thiolated hairpin-structured nucleotide probe is first inserted into the post lift-off region, and its complimentary nucleotide partner is thereafter introduced. Before the binding partner introduction, the fluorescence signal is quenched by the close distance of the dye to Au, where the subsequently observed high-contrast fluorescence image indicates sufficient surface space required in the binding partner recognition step. This result confirms that the diluted molecular matrix supported by the post lift-off region provides steric hindrance-free environment, which benefits in the recognition of other molecules onto the substrate. The third type of fabricated bioactive substrate lies on the anchoring of an active ligand, which can recognize a corresponding protein partner in the solution (Figure 2C). In order to minimize the protein nonspecific adsorption, an oligo(ethylene glycol) moiety containing TEG molecule is selected as the matrix material, which can also be lifted off by activated PDMS stamps in the CLL operation [21]. After the ligand–protein binding process, a sandwich-like assay is employed via the sequential attachment of primary and reporter-labelled secondary antibodies. This bulky assay design requires the spatial surrounding to give efficient recognition comparing to the previous two substrate types. It is clear that the fluorescence pattern created via this approach also presents very high contrast, indicating that a proper environment was provided by the post lift-off region even with bulky biological species. From the great capability of three bioactive substrate types toward diverse molecules and recognition processes under appropriate experimental condition adjustments, the great potential of CLL-treated surfaces for a variety of applications is ratified.

**Figure 3:**
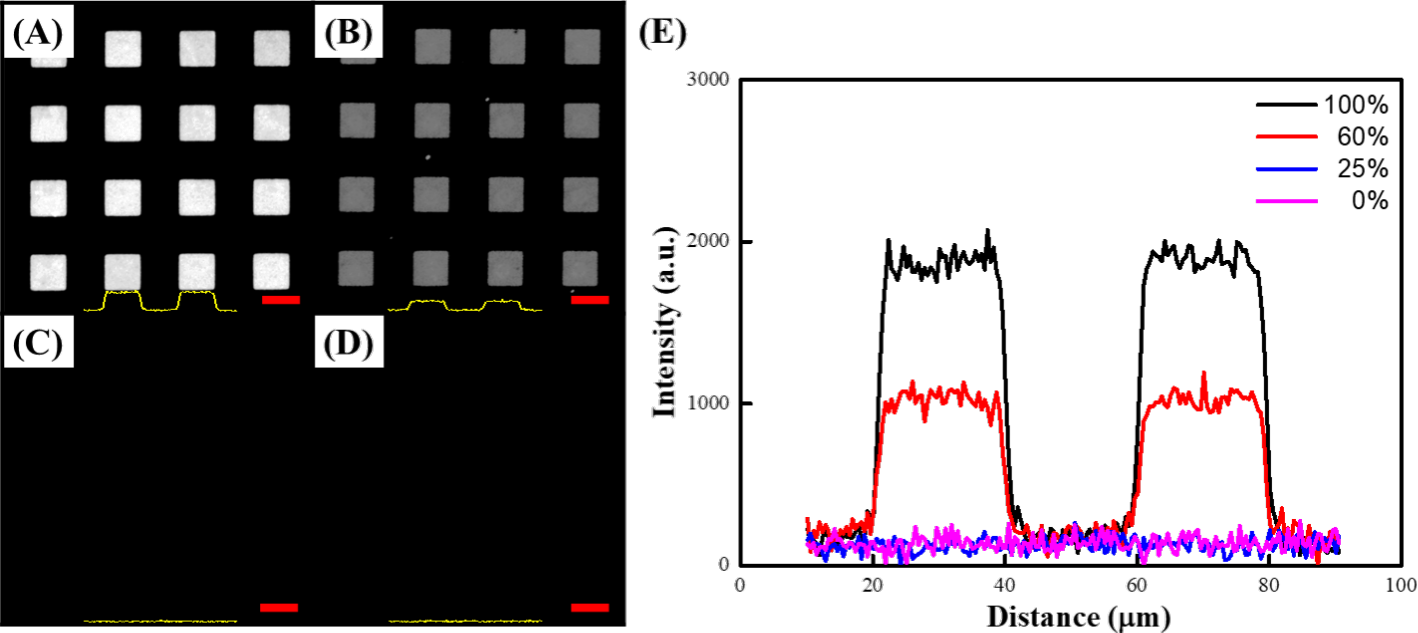
Fluorescence images (A–D) and intensity profiles (E) of FAM-labelled DNA-patterned surfaces fabricated by CLL for (A) 100% MCU SAM (0% UT), (B) 60% MCU SAM (40% UT), (C) 25% MCU SAM (75% UT), and (D) 0% MCU SAM (100% UT). The scale bars are 20 μm.

It should be noted that molecular matrix composition plays a key role in biological species selective recognition [14–15,33–34]. A different probe density requirement is therefore expected when diverse biocapturing environments are employed. Taking advantage of the straightforward CLL operation and convenience of alkanethiol SAM formation, the interface-contact-induced reaction can be applied to satisfy appropriate probe quantity control. As depicted in Figure 3, different percentages of hydroxy-group-terminated MCU and methyl-terminated UT molecules are utilized to form SAMs on Au. The substrates are thereafter treated by the CLL process and incubated with FAM-labelled nucleotide probes under the same conditions. It is clear to see that the probe-anchored area fluorescence intensity decreases along with the MCU percentage, which can be attributed to two presumable factors. First, the lack of a contact-induced reaction toward the activated PDMS surface of methyl-terminated alkanethiol diminishes the amount of lift-able Au thiolates in the matrix, resulting in reduced SAM defect creation in the CLL process. A consistent observation of decreased [Ru(NH_3_)_6_]^3+^ CV response along with larger redox peak separation Δ*E*_p_ (from 0.08 V to 0.15 V) under reduced MCU percentage also indicates less defects in the SAM environment (Figure S1, Supporting Information File 1). Second, the increase of hydrophobicity due to the presence of methyl-terminated alkanethiol leads to unfavorable hydrophilic biological probe insertion. The observed fluorescence signals are therefore reduced with the increase of UT molecule ratio in the matrix. It is important to note that although single-stranded DNA probes may adsorb nonspecifically on methyl-terminated SAMs [35], their contribution to fluorescence image contrast is deducted due to the close dye-to-Au distance induced nonradiative energy transfer. Given the controllable matrix composition and the ease of CLL operation, the surface probe quantity is well-tunable to satisfy the various needs of different biological platforms.

**Figure 4:**
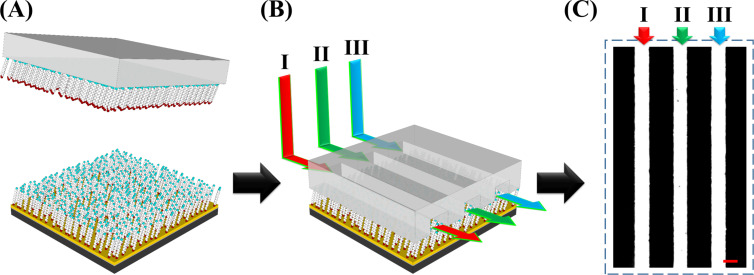
Schematic illustration of multiplexed bioactive surface fabrication by the combination of chemical lift-off lithography (CLL) and microfluidics: (A) CLL treatment with an activated featureless PDMS stamp. (B) Probe insertion by the combination of a microfluidic device. (C) The fluorescence image represents a multiplexed surface spatially anchored with different probes including (I) Hg^2+^-specific, (II) adenosine-specific, and (III) cocaine-specific probes. The scale bar is 20 μm.

The CLL-created unique molecular environment can also be extended to fabricate multiplexed bioactive arrays. As illustrated in Figure 4, a spatially addressed biological-probe-anchored substrate is fabricated via the combination of a microfluidic device and a CLL-treated surface. The diluted MCU alkanethiol matrix is first generated by applying activated featureless PDMS to interact with the whole SAM-modified Au surface followed by a subsequent lift-off step. This post lift-off surface is thereafter conformally sealed with a PDMS microfluidic device, rendering three 20 μm channels with 30 μm spacing in between. Three individual solutions containing different types of thiolated bioactive probes (targeting Hg^2+^, adenosine, and cocaine) are accordingly injected into these channels for a 60 min of probe insertion duration. Finally, the PDMS device is separated from the Au surface and a multiplexed bioactive-probe-anchored substrate is created. For demonstration, this spatially addressed bioactive substrate is tested with the signal reporter labelled probe direct insertion approach. It is found that three different molecules all insert into the desired position during fluidic incubation and give a multiplexed image with high fluorescence signal, as shown in Figure 4C. Channel I, II, and III are Hg^2+^, adenosine, and cocaine-specific probe anchored positions, respectively. To demonstrate the anchored probe’s selectivity toward its corresponding target, this multiplexed substrate was tested with a 1 mM adenosine solution. The obvious disappearance in the fluorescence signal in column II (Figure S2, Supporting Information File 1) points to the high platform selectivity toward its corresponding targets. The results indicate that CLL-fabricated substrates not only render high versatility for biological probes, but also entail great potential toward analytical technique integration. It is also important to note that biologically interesting closed-packed different chemical patterns on the same substrate could also be achieved via this approach. For example, the lift-able and reactive amine-terminated alkanethiol SAM-modified substrate can be integrated with a microfluidic device and functionalized with different moieties through various injected solutions. The device can thereafter be separated and the exposed post lift-off regions can be back-filled with a different chemical functionality. These multiplexed and closed-packed chemical patterns can therefore be utilized for an abundance of biological recognition applications [14,20,24].

**Figure 5:**
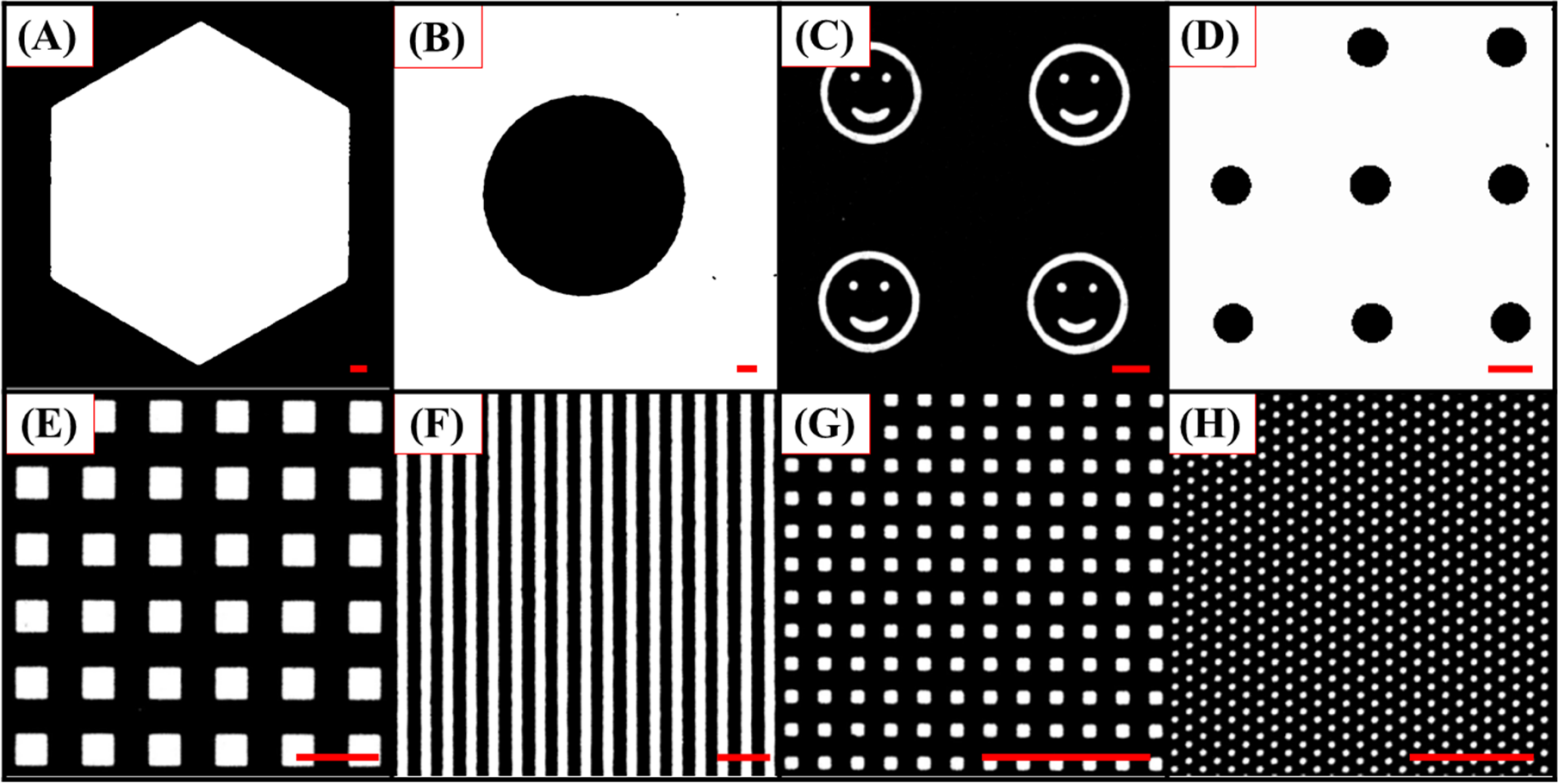
Fluorescence microscopy images (A–H) of FAM-labelled DNA patterned surfaces with various features and sizes created by CLL. The scale bars are 50 μm.

**Figure 6:**
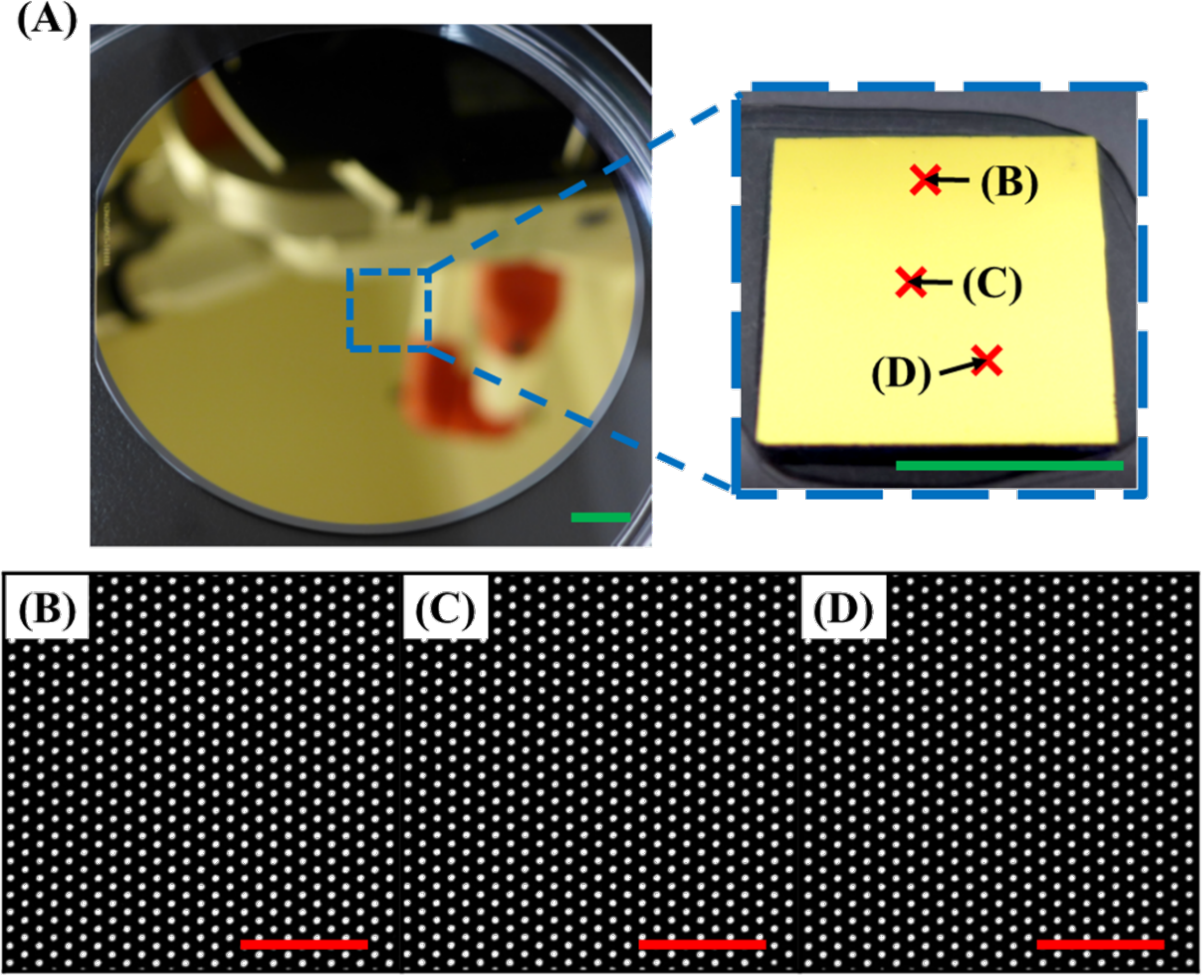
A representative photo (A) and fluorescence images (B–D) of the large-area bioactive surface fabricated by CLL. (A) Photo images showing a wafer-scale patterning achieved by CLL. (B–D) Fluorescence images obtained from different sampling spots indicated in (A) on the same surface. The green and red scale bars are 1.0 cm and 20 μm, respectively.

Because the bioactive surface feature size and geometry may affect the substrate’s capability toward practical application and the functionality of the anchored probes, the CLL-based fabrication process was tested to create surfaces with a variety of molecule arrangements in different dimensions. As shown in Figure 5, different PDMS stamps rendering diverse geometries, either with protruding (Figure 5A,C,E,G, and H) or depressed (Figure 5B,D, and F) features, were applied on the SAM-modified Au surface to create molecular matrix patterns. With the adoption of a simply operated signal reporter labelled probe insertion strategy, high-contrast fluorescence images with versatile geometries are obtained and no obvious signal output fluctuation is observed with the different sizes. It should be noted that bioactive substrates created by the CLL process can also be made very uniform over a large area. As demonstrated in Figure 6, fluorescence images obtained from distinct sampling spots on a 4 inch silicon wafer substrate represent the same results with minimized signal fluctuation. The high repeatability and scale up capability of fabricated biologically active patterns confirms the technique’s potential toward practical applications.

## Conclusion

The chemical lift-off process enables fabrication of diverse bioactive substrates in a straightforward manner. The approach generates abundant alkanethiol SAM defects via interface-contact-induced reactions between the activated PDMS stamp and hydroxy-group-terminated SAM molecules. The separation of these two surfaces leads to the creation of molecular level matrix defects, which enables the subsequent bioactive probe anchoring. This CLL-based substrate is capable of conjugating a variety of biological species, such as nucleotide-tethered probes, orientation changeable molecules, and bulky proteins or antibodies. It is found that these surface-tethered probes maintain their biological activity and the fabricated pattern sizes and geometries are well-tunable. In addition, the creation of multiplexed arrays can also be accomplished by the integration of the CLL process with a microfluidic device, indicating the great potential of this strategy toward practical bioapplications. The observed bioactive substrate properties are attributed to the unique diluted SAM environment created by the CLL-process-induced Au-thiolate rupture happening at the interface. Due to the randomly distributed thiol molecule residual generated during the process, a diluted matrix rendering copious SAM defects is expected. This environment is therefore able to support the tethering of biological probes with controllable density, and offers sufficient space for biorecognition under the adjustment of experimental conditions. We believe that this CLL-treated surface can be used in conjunction with various biomolecules and a convenient approach to fabricate large-scale bioactive substrates with well-defined patterns is anticipated.

## Supporting Information

File 1 Additional information.
