# Supplementary material for: Wafer-scale bioactive substrate patterning by chemical lift-off lithography
Source: Beilstein J Nanotechnol. 2018 Jan 26;9:311–20. doi: 10.3762/bjnano.9.31 (PMC5789397; doi:10.3762/bjnano.9.31)
Supplement: File 1 — Additional information. [file Beilstein_J_Nanotechnol-09-311-s001.pdf]

Supporting Information

for

Wafer-scale bioactive substrate patterning by chemical lift-off lithography

Chong-You Chen, Chang-Ming Wang, Hsiang-Hua Li, Hong-Hseng Chan, and Wei-Ssu Liao*

Address: Department of Chemistry, National Taiwan University, Taipei 10617, Taiwan

Email: Wei-Ssu Liao - wsliaochem@ntu.edu.tw

*Corresponding author

Additional information

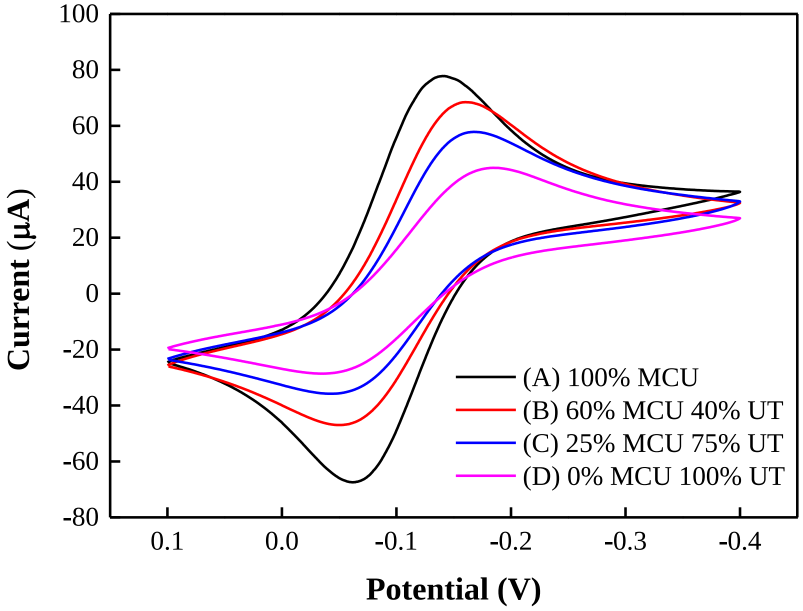

Figure S1: Cyclic voltammograms of 1 mM $[Ru(NH_3)_6]^{3+}$ on different CLL-treated substrates when (A) 100% MCU SAM (0% UT), (B) 60% MCU SAM (40% UT), (C) 25% MCU SAM (75% UT), and (D) 0% MCU SAM (100% UT) were used as the lift-off matrix. The measurements were performed in 25 mM Tris buffer at pH 7.4, and the scan rate was 100 mV/s.

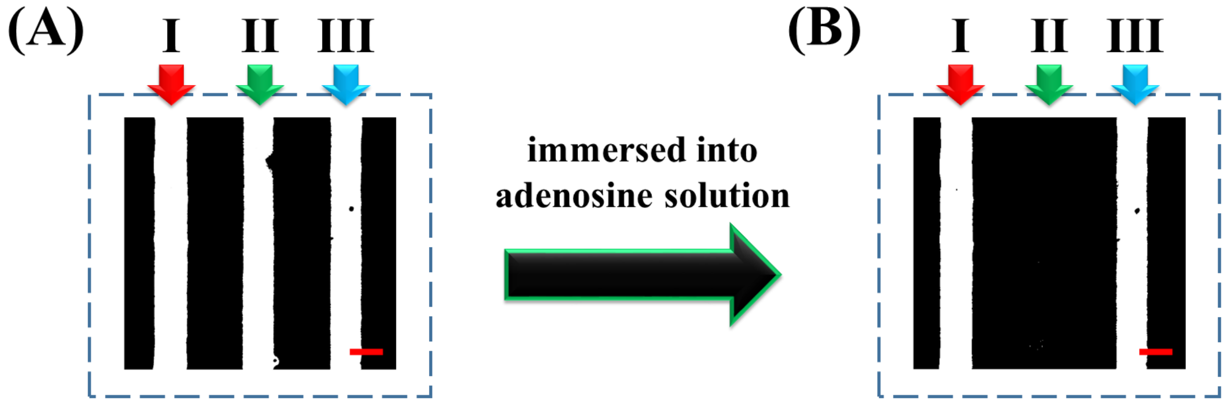

Figure S2: The CLL-created multiplexed surface tethered with three different types of DNA probes: I: Hg^{2+}-specific, II: adenosine-specific, III: cocaine-specific. The column II fluorescence signal clearly disappears upon the addition of 1 mM adenosine solution. The scale bars are 20 μm.
